# Obstetric Memoranda

**Published:** 1877-04-20

**Authors:** 


### OBSTETRIC MEMORANDA.

This case may be given in brief detail. Mrs. W——, æt. 32, seventh pregnancy at full term ; previous labors prolonged and difficult, pains very severe and prostrating. For some months before delivery, the lower extremities were much swollen with dropsical effusion, and there was general œdema of the body and face especially about the eyes. She was also troubled with severe bronchitis, for which I treated her with good effect during the last three weeks preceding delivery.

She was in a very despondent condition, fearing the worst. The heart's action was very weak.

I was called to her at 6:10 P. M. She had had strong and regular labor pains, from about 1 P. M., and suffered from sharp, premonitory ones, from the preceding evening. On examination, I found the pelvic cavity roomy, the passages cool and moist, the rectum empty, the patient passing water freely, the pains strong, regular, and succeeding one another at rapid intervals. The head was presenting, the waters were unbroken, and the "os" was dilated to something less than the size of shilling ; its edges were very thin, hard, sharp, resisting and undilatable. I at once gave a dose of chloral, and followed up with two others, at intervals of twenty minutes. I gave in all sixty grains. Its good effects were soon apparent.

These effects were,however, even more marked, as the progress of the case was very rapid. She enjoyed the same quiet deep sleep during the intervals between the pains, and felt herself greatly refreshed. The pains themselves were not really so intense, although the contractions were very strong. The child was born at 8:20 P.M., two hours after the administration of the first dose of chloral. It was a male of very large size. The anterior lip of the uterus got caught between the symphysis pubis and child's head. I had to push it up, and keep it there until the latter had descended sufficiently into the cavity of the pelvis, to prevent of its prolapse. The pulse remained at 84 beats to the minute. The post-partum contraction of the womb was good, expelling the placenta at the end of ten minutes, unaided by pressure. There was no post-partum hemorrhage. The patient felt most grateful, asserting that she had never had such an easy time of it in all of her previous confinements. She declared the effects of the chloral to be most soothing and refreshing.

From the foregoing case it is evident that chloral:

1. Had a strong controlling influence over the amount of pain felt.

2. That it secures quiet, refreshing sleep during the intervals of pain, thus husbanding the patient's strength.

3. That it has no retarding influence upon the progress of labor.

4. On the contrary, it seems to have a decidedly beneficial effect in steadying, and directing to a favorable issue, the uterine contractions; also in hastening the dilatation of the os uteri.

5. It does not in any way weaken post-partum uterine contraction.

From my experience, it is of great service, where, from idiosyncrasy on the part of the patient, the smallest dose of opium could not possibly be given.—*Medical Press and Circular.*

